# Citrus Flavonoids Supplementation as an Alternative to Replace Zinc Oxide in Weanling Pigs’ Diets Minimizing the Use of Antibiotics

**DOI:** 10.3390/ani13060967

**Published:** 2023-03-07

**Authors:** Montserrat Paniagua, Sandra Villagómez-Estrada, Francisco Javier Crespo, José Francisco Pérez, Anna Arís, Maria Devant, David Solà-Oriol

**Affiliations:** 1 Technical and R&D Department, Quimidroga S.A., 08006 Barcelona, Spain; 2 Faculty of Veterinary, Escuela Superior Politécnica de Chimborazo, Riobamba 060155, Ecuador; 3 Animal Nutrition and Welfare Service (SNIBA), Department of Animal and Food Science, Autonomous University of Barcelona, 08193 Barcelona, Spain; 4 R&D Department, HealthTech Bio Actives (HTBA), Sociedad de Responsabilidad Limitada Unipersonal (S.L.U.), 08029 Barcelona, Spain; 5 Ruminant Production, IRTA (Institut de Recerca i Tecnologia Agroalimentàries), Torre Marimon, 08140 Caldes de Montbui, Spain

**Keywords:** citrus flavonoid, antibiotic use, bitter taste receptors, gene expression, gut health, weaned piglet

## Abstract

**Simple Summary:**

Weaning is a stressful period for pigs that causes gastrointestinal disruption and low growth rates. For a long time, zinc oxide at pharmacological doses, along with different antibiotics, has been used prophylactically during this phase to reduce the incidence of these gastrointestinal problems. Nowadays, the increasing concern about the environment, along with the global and constant growth of bacterial resistance to antibiotics, has led to the prohibition of the prophylactic use of zinc oxide in diets for piglets in the EU, along with the implementation of new regulations on the use of antibiotics. Consequently, the pig sector faces the important challenge that supposes developing alternatives to the classical system based on the use of these antimicrobial compounds. This study is the first step to achieving this goal by minimizing the use of various antibiotics and zinc oxide in weanling pigs by supplementing citrus flavonoids and only one antibiotic (amoxicillin). Accordingly, the influence of zinc oxide plus antibiotics and citrus flavonoids plus amoxicillin in weaned pigs has been investigated and its impact on growth performance, gut microbiology profile, gut signaling, intestinal architecture, and serum biomarkers indicative of stress and inflammatory responses have been evaluated. Citrus flavonoids plus amoxicillin improved growth performance and gut health, evidencing a positive microbial modulation, stress status reduction, and a positive effect on the gastrointestinal barrier, and other digestive functions. Additionally, the expression of some bitter taste receptors in the intestine has been increased when supplementing both dietary strategies, the one based on zinc oxide or the one based on citrus flavonoids supplementation. Consequently, the present study shows that in weanling piglets, the supplement of citrus flavonoids with amoxicillin might be a promising alternative to the dietary use of pharmacological doses of zinc oxide with more than two antibiotics, therefore minimizing the use of antimicrobial compounds without detrimental effects on performance.

**Abstract:**

Since citrus flavonoids have antioxidant and anti-inflammatory properties, it was hypothesized that these compounds would become a suitable alternative to the use of therapeutic doses of zinc oxide at weaning. A total of 252 weaned pigs ([LargeWhite × Landrace] × Pietrain) were distributed according to BW (5.7 kg ± 0.76) into 18 pens (6 pens per diet, 14 pigs/pen). Three experimental diets for the prestarter (0–14 d postweaning) and starter (15–35 d postweaning) period were prepared: (i) a nonmedicated (CON) diet, (ii) a CON diet supplemented with zinc oxide at 2500 mg/kg, amoxicillin at 0.3 mg/kg and apramycin at 0.1 mg/kg (ZnO), and (iii) CON diet with the addition of a commercial citrus flavonoid extract at 0.3 mg/kg and amoxicillin at 0.3 mg/kg (FLAV). Pig BW, ADG, ADFI, and FCR were assessed on d7, d14, and d35. Samples of intestinal tissue, cecal content, and serum were collected on day seven (18 piglets). FLAV treatment achieved greater BW and ADG during the starter and for the entire experimental period compared with the CON diet (*p* < 0.05), whereas ZnO pigs evidenced intermediate results. Jejunum tissue analysis showed that pigs fed the FLAV diet overexpressed genes related to barrier function, digestive enzymes, and nutrient transport compared to those pigs fed the CON diet (*p* < 0.05). An increase in the abundance of bacterial genera such as *Succinivibrio*, *Turicibacter*, and *Mitsuokella* (*p* < 0.05) was observed in the FLAV compared with the CON and ZnO piglets. ZnO and FLAV increased the expression of TAS2R39, while ZnO pigs also expressed greater TAS2R16 than CON (*p* < 0.05) in the intestine. FLAV treatment improved the gut function, possibly explaining a higher performance at the end of the nursery period. Consequently, citrus flavonoids supplementation, together with amoxicillin, is a promising alternative to the use of zinc oxide plus amoxicillin and apramycin in weanling pigs, minimizing the use of antibiotics.

## 1. Introduction

Among livestock production, the swine industry provides one of the most important protein sources for human nutrition [1]. Even though in recent decades a high degree of improvement has been achieved at the genetic level (prolificity, efficiency, and performance), paradoxically, the weaning phase continues to be a critical period within this system. Indeed, the improvement in a sows’ prolificity has led to a reduction in the birthweight of neonates, also increasing litter heterogeneity with serious consequences during postnatal life [2]. For decades, several antimicrobial compounds, such as zinc oxide at pharmacological doses and antibiotics as growth promoters, have been widely used as a practical strategy to reduce and control postweaning syndrome in pigs. For instance, zinc oxide possesses a multifactorial mode of action, improving digestion and nutrient digestibility, acting as an antioxidant and immunomodulatory molecule with antibacterial effects and, consequently, impacting positively on intestinal morphology and health [3]. However, antibiotics and zinc oxide are involved in the continuous increase of antibiotic-resistant bacteria and, even more, zinc oxide is the direct cause of negative effects polluting the environment [4]. Therefore, accordingly to the worldwide cross-sectorial strategy for “One Health” fixed by the FAO, the OIE, and the WHO, the use of antibiotics should be controlled and restricted. Meanwhile, zinc oxide at pharmacological doses has been completely banned in the European Union since June 2022 [5].

Different feed additives have been studied and proposed as an alternative to zinc oxide to improve postweaning pigs’ health and reduce the incidence of diarrhea [3]. Citrus flavonoids possess antioxidant and anti-inflammatory properties, and different studies have evidenced their effects as antimicrobial and immunomodulatory molecules [6,7,8]. Additionally, citrus flavonoids and their metabolites have shown beneficial effects on the intestinal barrier through different mechanisms of action [8]. Citrus flavonoids, as bitter compounds, modify the gene expression of bitter taste receptors (TAS2R), similar to other compounds with antimicrobial, anti-inflammatory, and antioxidant capacity [9], and some bitter compounds have shown promising effects to substitute zinc oxide at pharmacological doses [10]. Therefore, it was hypothesized that citrus flavonoids would become a suitable alternative to the use of therapeutic doses of zinc oxide, also reducing the use of antibiotics in weanling pigs. The objective of this study was to evaluate the effect of supplementing citrus flavonoids and amoxicillin in replacing the use of pharmacological doses of zinc oxide together with several antibiotics in weanling piglets as a first step in the demedicalization process of this productive phase. Accordingly, gene expression, microbiota, and histomorphology in the guts of weanling pigs, along with performance parameters and stress markers, were studied to elucidate the potential application of citrus flavonoids in weanling pigs.

The experiment was planned, designed, and conducted at the end of the year 2018, aiming to deal with the reduction of antimicrobials in swine production (antibiotics and zinc oxide) and facing a new scenario and anticipating the ban on zinc oxide in 2022.

## 2. Materials and Methods

### 2.1. Ethics Statement

Experimental procedures were approved by the Ethics Committee of the Universitat Autònoma de Barcelona (approval code CEEAH2788M2) based on the European Union guidelines for the use of animals in research [11].

### 2.2. Animals and Housing

At weaning (21 ± 1.6 d), 252 pigs ((Large White × Landrace) × Pietrain) with an initial average BW of 5.7 ± 0.76 kg were used in a 35-d study. Animals were identified through an ear tag, individually weighted, blocked according to the initial BW, and distributed into three experimental diets in 18 pens (6 pens per treatment, 14 pigs per pen) within the same weanling room. Each pen (3.12 m^2^) allotted entire males and females. The commercial weaned facility owned by the Universitat Autònoma de Barcelona had a fully slatted floor, unlidded hoppers (TR5, Rotecna, Spain), and nipple drinkers. Temperature and ventilation rates were monitored using thermostatic heaters and exhaust fans adjusted depending on the age of the pigs (28 to 22 °C).

### 2.3. Experimental Design and Dietary Treatments

Two-phase diets (Table 1) were formulated to meet or exceed nutrient requirements [12]: the prestarter (PS) phase from days 1 to 14 and the starter (ST) from days 14 to 35. Three experimental diets were prepared according to the supplementation or not of antimicrobial and experimental products. For the PS feed phase, a nonmedicated (CON; no antibiotic or pharmacological levels of zinc oxide) was used as the basal diet for all experimental diets. The positive control diet (ZnO) consisted of the addition of zinc oxide at 2500 mg/kg, amoxicillin at 0.3 mg/kg, and apramycin at 0.1 mg/kg products to the CON diet. The third diet consisted of the CON diet plus the addition of a commercial citrus flavonoid product (Bioflavex^®^, HTBA, Barcelona, Spain) at 0.3 mg/kg and amoxicillin at 0.3 mg/kg (FLAV). During the ST phase, diets remained the same except for the ZnO diet. It consisted of the addition of zinc oxide at 1500 mg/kg plus the addition of amoxicillin at 0.3 mg/kg, neomycin at 0.19 mg/kg, and tiamulin at 0.10 mg/kg. The antimicrobial plan used in the positive control was applied based on the in-farm therapeutic strategy already conducted in the commercial conditions according to the health status and inherent challenges (common clinical signs of postweaning diarrhea associated with ETEC *E. coli*, Streptococcal meningitis, and swine dysentery at the second phase of the nursery period). In the FLAV treatment, amoxicillin was used throughout the study due to the broad spectrum of this antibiotic and the high prevalence of Streptococcal meningitis on the farm. 

For each dietary treatment, the feed was offered in mash and pellet form during the PS and ST phases, respectively. Both feed and water were provided ad libitum.

### 2.4. Experimental Procedures and Sampling

In order to calculate the average daily gain (ADG), average daily feed intake (ADFI), and feed conversion ratio (FCR), the individual BW of the pigs, as well as feed disappearance from each feeder, was recorded on days 7, 14, and 35. Pigs were daily monitored for mortality and illness incidents.

#### 2.4.1. Feed Analysis

Proximate analytical determination of diets was performed following the Association of Official Agricultural Chemists International (2005) methods: dry matter (AOAC 934.01), crude protein (AOAC 968.06), ether extract (AOAC 920.39), and ash (AOAC 942.05). Neutral detergent fiber content was determined using the Ankom nylon bag technique (Ankom 200 fiber analyzer, Ankom Technology, Macedon, NY, USA).

#### 2.4.2. Proinflammatory and Stress Markers

From each pen, three pigs (average BW) were selected to take blood samples on days 0, 7, and 14 of the trial. The same animal was sampled during the experimental period. Samples were taken by jugular puncture and collected in an individual vacutainer tube without anticoagulant (10 mL). Tubes were subsequently centrifuged at 1500× *g* for 10 min to obtain serum. Aliquots of serum were stored at −80 °C until chemical analyses. A PigMap analysis was performed on the Olympus AU 400 analyzer [13]. Two commercial ELISA kits were utilized to analyze cortisol (DRG, Marburg, Germany) and porcine TNF-alpha (R&D Systems, Abingdon, UK) content.

#### 2.4.3. Histomorphological Analysis

On day 7, samples of the jejunum and ileum tissue (about 5 cm) were collected from one pig per pen (*n* = 6). Selected animals exemplified the average pig BW within the pen. Pigs were sedated using zolazepam and xylazine and subsequently euthanized by an overdose of pentobarbital. Samples were fixed in 4% paraformaldehyde and then embedded in paraffin. Then, sections were stained with a hematoxylin-eosin solution. Villus height, crypt depth, villus height to relative crypt depth ratio (V:C), and the number of goblet cells and lymphocytes were measured by using a light microscope. Only full and vertical-oriented villi were considered within the analysis.

#### 2.4.4. Gene Expression Analysis

From the same jejunum and ileum sections, samples of about 1 cm were collected. Intestinal samples were rinsed in phosphate-buffered saline and immediately snap frozen in 1 mL of RNAlater (Deltalab, Rubí, Spain). Samples were stored at −80 °C until analysis. An Open Array Real-Time PRC Platform (Applied Biosystems, Waltham, MA, USA) was self-designed as described by González-Solé [14]. Briefly, extensive literature research was accomplished to finally select 56 genes. The main criteria for selection were the involvement of the gene on physiological functions such as immune response, barrier function, digestion processes, nutrient transport, and stress responses. Sample procedures, data collection software, and sample quality analysis were executed as previously defined by Villagómez-Estrada [15].

In addition, four genes related to bitter taste receptors were individually analyzed (TAS2R7, TAS2R16, TAS2R38, and TAS2R39) from jejunum and ileum samples. RNA was extracted by homogenizing tissues in TRIzol (Invitrogen, Waltham, MA, USA) using a Polytron Instrument (IKA, Königswinter, Germany). PrimeScript RT Reagent Kit (Takara, Frankfurt, Germany) was used to transcribe RNA to cDNA, following the manufacturer’s instructions. The quality of RNA was assessed by a NanoDrop instrument (ThermoFisher, Madrid, Spain) at 260, 280, and 230 nm. Quantification of gene expression was performed as described in Paniagua et al. [9].

#### 2.4.5. Microbial Molecular Analysis

On day 7, from the same selected animals, cecal content (500 mg) was collected for 16-S microbial sequencing analysis. Samples were immediately frozen and stored at −80 °C until analysis. Bacterial DNA was obtained by following the manufacturer’s instructions for the commercial QIAamp DNA Stool Min Kit (Qiagen, West Sussex, UK). After the proper verification of DNA concentration and purity (NanoDrop 1000 Spectrophotometer; Termo Fisher, Wilmington, DE, USA) it was eluted in 200 μL of Qiagen buffer AE ad stored at −80 °C until analysis. Samples were amplified (500 cycles) using the MiSeq^®^ Reagent Kit v2 (MiSeq, Illumina, San Diego, CA, USA) for the V3–V4 regions of the bacteria. Sequence reads were processed on the QIIME v.1.9.1 pipeline [16] and clustered to operational taxonomic units (OTUs) at a 97% sequence similarity. Reads were selected by the subsampling open reference approach at 10% of sequences subsampled [17] and assigned to a taxonomy using 16S GreenGenes v.13.8 reference database [18] at a 90% confidence threshold. OTUs with a relative abundance across all samples lower than 0.005% were eliminated [19].

#### 2.4.6. Statistical Analysis

Prior to ANOVA analysis, the normality and homogeneity of the data were assessed using the Shapiro–Wilk test. Data were analyzed through the MIXED procedure of SAS (version 9.4, SAS Institute, Cary, NC, USA) considering a randomized complete block design. The effect of the experimental diet was considered a fixed effect, whereas the BW block counted as a random effect. The pen was the experimental unit. In the case of acute phase proteins, histomorphological measurements, gene expression, and microbiota community data corresponded to the individual pig selected that represented the average BW in each pen, therefore the animal represented the pen (the experimental unit). For acute phase proteins and stress markers, the initial measurements (d 0) were used as covariables. Digesta bacteria diversity was analyzed at the OTU level using a vegan package [20]. Shannon and Inverse Simpson estimators were used to assess the alpha diversity on the basis of raw counts. While beta diversity dissimilarities were evaluated by multivariate ANOVA using the Adonis function. An ANOVA test was performed to test the experimental group’s differences in microbial richness and diversity. The zero-inflated log-normal mixture model was considered by the differential abundance analysis.

Biostatistical analysis of gene expression and microbiota community data was performed in R Studio v.3.5.1 software. The means and SEM values for bitter taste gene expression data presented in the figures correspond to nontransformed data and *p*-values to those obtained by an ANOVA of transformed data. They were analyzed using a mixed-effects model (version 9.2, SAS Inst.Inc., Cary, NC, USA), where the model included treatment, tissue, and the interaction between treatment and tissue as fixed effects. For both, gene expression and microbiota analysis, the Benjamini and Hochberg false discovery rate (FDR) multiple testing correction was performed [21]. Significance was declared at a probability of *p* ≤ 0.05 and tendencies were considered when the *p*-value was between >0.05 and <0.10.

## 3. Results

### 3.1. Growth Performance

Pig performance response for the experimental diets is shown in Table 2. Feeding the FLAV diet tended to increase the pigs’ BW at day seven when compared to CON animals (*p <* 0.10). Interestingly, pigs from the FLAV treatment achieved greater BW and ADG during the ST and for the entire experimental period compared to the CON diet (*p* < 0.05). However, BW and ADG from the ZnO animals showed no differences when compared to CON and FLAV treatments at the end of the study. Although both treatments, FLAV and ZnO, improved ADFI during the ST and for the entire experimental period, no influence of experimental diets was observed on FCR (*p* > 0.10).

Mortality was 1.59% and was not related to any dietary treatment (*p* = 0.752).

### 3.2. Gene Expression Analysis

**Figure 1:**
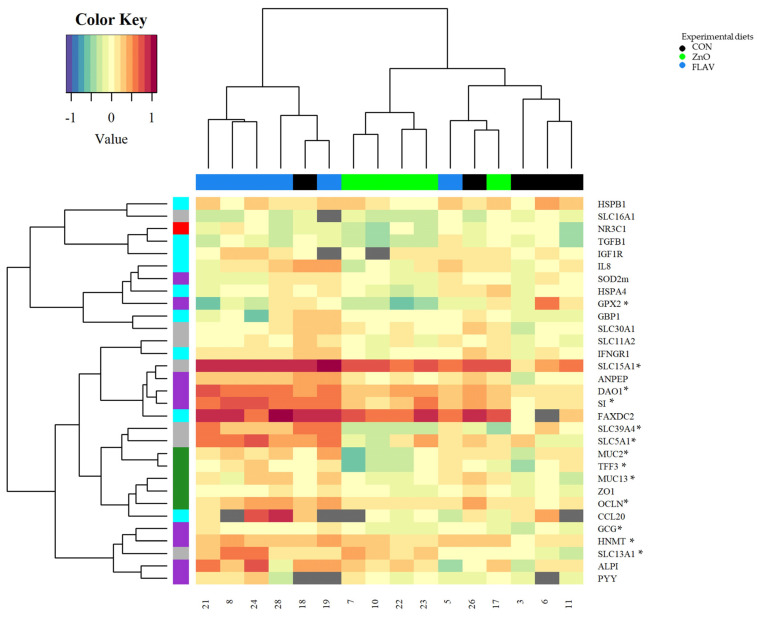
Heatmap and hierarchical clustering of jejunum gene expression levels of pigs fed experimental diets. Significant differences are indicated using an asterisk symbol (*p* < 0.10). Data are means of 3 pigs at 7d postweaning.

**Figure 2:**
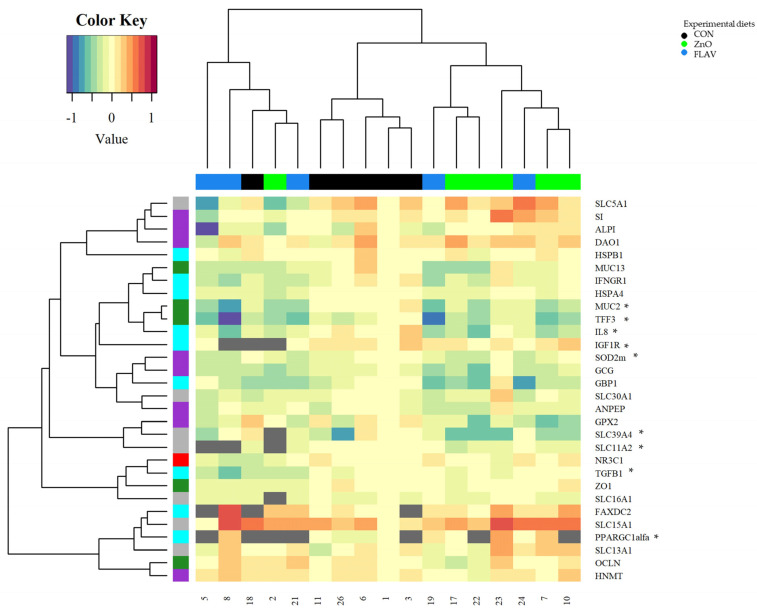
Heatmap and hierarchical clustering of ileum gene expression levels of pigs fed experimental diets. Significant differences are indicated using an asterisk symbol (*p* < 0.10). Data are means of 6 replicate pens with 1 sampled pig per experimental diet.

For a complimentary evaluation of the experimental diets’ effects on pig physiological status, a gene expression analysis of jejunum and ileum tissue was performed on day seven postweaning (Figure 1 and Figure 2). A total of 46 genes were successfully amplified, though only up to 12 genes significantly differed among the experimental diets (*p* < 0.05; Table 3 and Table 4). Gene expression differed between intestinal tissues. The analysis of jejunum tissue showed dissimilarities in gene expression compared to ileum tissue. Pigs fed the FLAV diet overexpressed genes related to barrier function (MUC13), digestive enzyme (DAO1, GCG, HNMT, and SI), and nutrient transport (SLC13A1 and SLC15A1) compared to those pigs fed the CON diet (*p* < 0.05; Table 1). Interestingly, pigs the fed ZnO diet showed downregulation of the Zn transporter gene (SLC39A4) compared to pigs fed the CON and FLAV diets (*p* < 0.0001). Ileum analysis showed that pigs fed the CON diet had an upregulation of genes involved in barrier function (MUC2 and TFF3), inflammatory response (IFNGR1 and IL8), and antioxidant enzymes (GPX2 and SOD2m) but a downregulation of the PPARGC1α gene (immune response) compared to the FLAV diet (*p* < 0.05; Table 3). However, the CON pigs had downregulation of some nutrient transport genes (SLC11A2 and SLC39A4) than the ZnO pigs (*p* < 0.05; Table 4).

### 3.3. Bitter Taste Receptors

**Figure 3:**
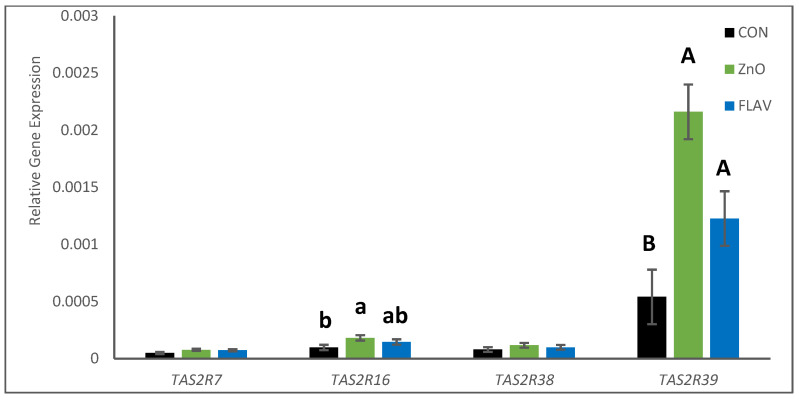
Gene expression of TAS2R on pigs fed the experimental diets analyzed by experimental diet. a, b means statistically different (*p* < 0.05) within TAS2R16; A, B means statistically different (*p* < 0.05) within TAS2R39.

**Figure 4:**
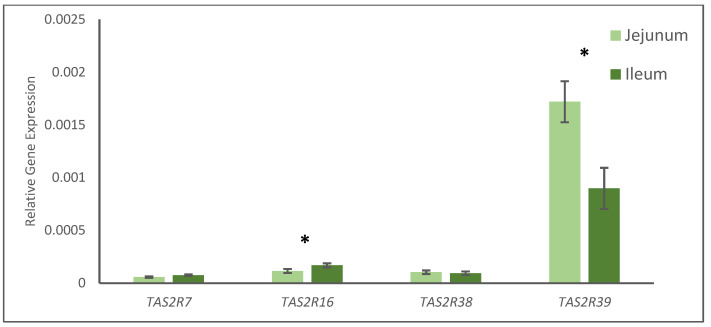
Gene expression of TAS2R of pigs fed the experimental diets analyzed by tissue. * Means statistically different (*p* < 0.05).

Regarding the TAS2R gene, no interaction was observed between experimental diets and tissues for the different receptors studied. The gene expression of TAS2R7 and TAS2R38 was not affected either by diet or by tissue. Nonetheless, the effect of diet and tissue was observed on TAS2R16 and TAS2R39 gene expression (Figure 3 and Figure 4).

Pigs supplemented with zinc oxide had a significant increase in the gene expression of TAS2R16 (*p* < 0.05; Figure 3) compared to the CON animals, and the expression of this receptor was greater in the ileum than in jejunum samples (*p* < 0.05; Figure 4), regardless of the experimental diet. Additionally, FLAV and ZnO diets increased the gene expression of TAS2R39 compared with the CON pigs (*p* < 0.05; Figure 3), whereas TAS2R39 was less expressed in the ileum than in the jejunum (*p* < 0.05; Figure 4).

### 3.4. Microbial Molecular Analysis

**Figure 5:**
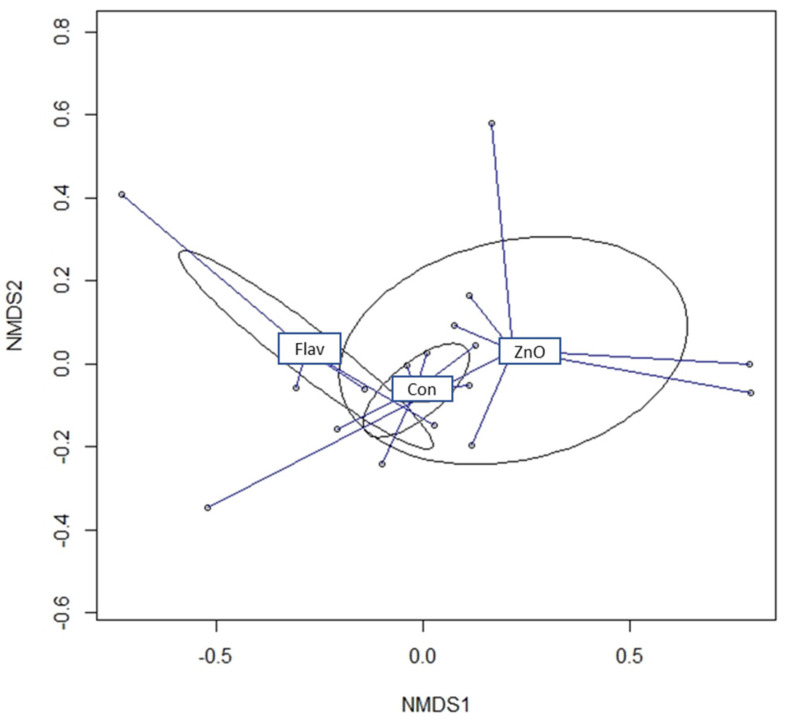
Nonmetric dimensional scaling (NMDS) plot of dissimilarity matrix based on Bray–Curtis distance clustered (PADONIS = 0.023).

No effect of the experimental diet was observed for alpha estimators (*p* > 0.10; Table 5). Beta diversity analysis revealed distances between clustered samples of the FLAV and CON groups (PADONIS = 0.023; Figure 5).

**Figure 6:**
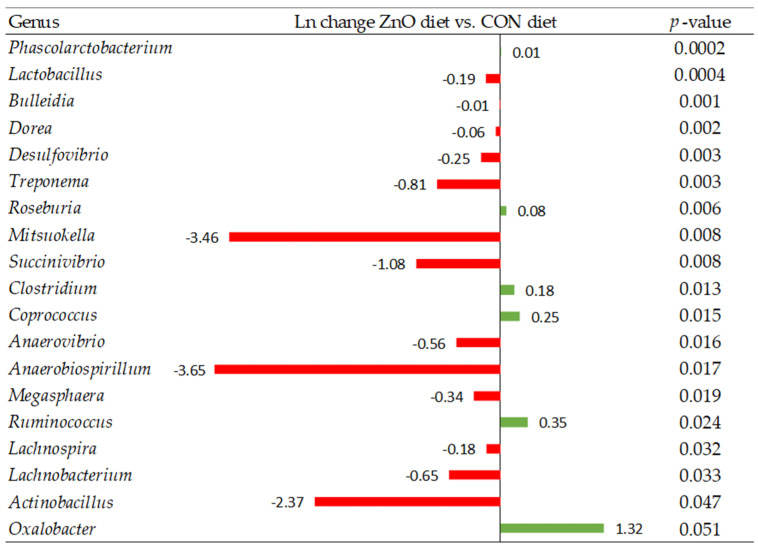
Differentially abundant taxa (Ln change) between diets supplemented with antimicrobial compounds (ZnO) and control diet (CON). Positive values (green color) and negative values (red color) indicate greater and lower abundance, respectively. Taxa are sorted by level of significance (from higher to lower). Only significant taxa are presented (*p*-value < 0.10). Data are means of 6 replicate pens for the main effect of level (one pig per replicate pen was sampled).

**Figure 7:**
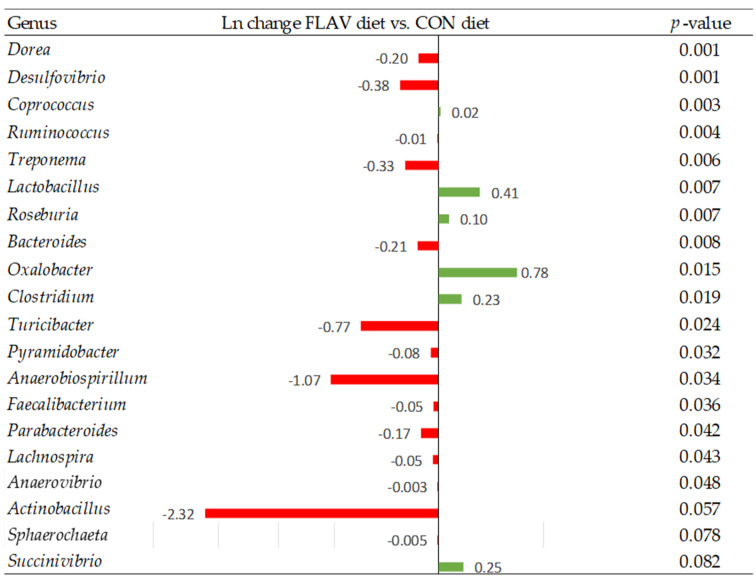
Differentially abundant taxa (Ln change) between diets supplemented with the flavonoid compound (FLAV) and the control diet (CON). Positive values (green color) and negative values (red color) indicate greater and lower abundance, respectively. Taxa are sorted by level of significance (from higher to lower). Only significant taxa are presented (*p*-value < 0.10). Data are means of 6 replicate pens for the main effect of level (one pig per replicate pen was sampled).

**Figure 8:**
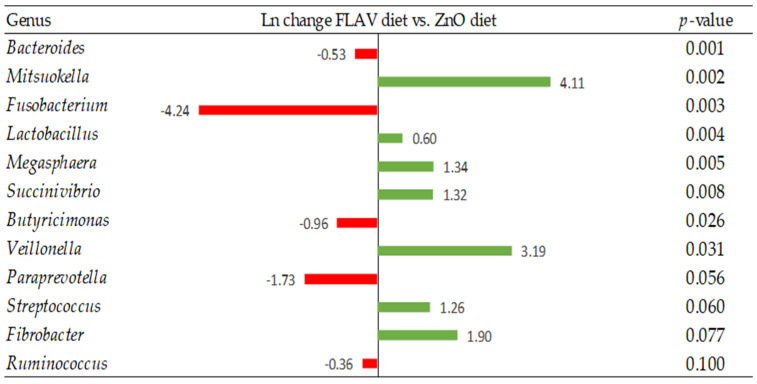
Differentially abundant taxa (Ln change) between a diet supplemented with antimicrobial compounds (ZnO) and a diet supplemented with flavonoid compounds (FLAV). Positive values (green color) and negative values (red color) indicate greater and lower abundance, respectively. Taxa are sorted by level of significance (from higher to lower). Only significant taxa are presented (*p*-value < 0.10). Data are means of 6 replicate pens for the main effect of level (one pig per replicate pen was sampled).

At the genus level, when pig diets were supplemented with zinc oxide, there was a decrease in the relative abundance of several genera such as *Lactobacillus*, *Desulfovibrio*, *Anaerovibrio*, *Lachnospira*, *Lachnobacterium*, and *Actinobacillus* compared to the CON diet (*p* < 0.05; Figure 6). Whereas, when the FLAV diet was offered to pigs the relative abundance of genera such as *Dorea*, *Desulfovibrio*, and *Actinobacillus*, among others, decreased compared to the CON diet (*p* < 0.05; Figure 7). Interestingly, the abundance of several genera of bacteria such as *Lactobacillus*, *Roseburia*, and *Clostridium* increased compared to the CON diet (*p* < 0.05; Figure 7). Comparing the FLAV diet to the ZnO diet, an increased abundance of *Mitsuokella*, *Lactobacillus*, *Megasphaera*, *Succinivibrio*, *Veillonella*, *Streptococcus*, and *Fibrobacter (p* < 0.10; Figure 8) was observed.

### 3.5. Intestine Histomorphometry Measurements

Ileum histomorphometry parameters are shown in Table 6. No effect of experimental diets on ileum histomorphometry parameters was observed except for a tendency on villus height (*p* = 0.094) and villus height to crypt depth V:C ratio (*p* = 0.059). Pigs fed the ZnO diet tended to have longer ileum villi and a higher V:C ratio than those fed the CON diet.

### 3.6. ProInflammatory and Stress Markers

As shown in Table 7, serum cortisol levels measured at day seven postweaning were lower in pigs fed the FLAV diet compared to those fed the ZnO diet (11.2 vs. 25.1 ng/mL; *p* = 0.007). No influence of experimental diets was observed on serum TNF-alpha and PigMap levels (*p* > 0.10).

## 4. Discussion

The main objective of this study was to explore a commercial approach to minimize the use of multiple antimicrobial substances in weanling diets, such as zinc oxide and antibiotics, through supplementation with citrus flavonoids. As the trial was performed under commercial farm conditions, the FLAV treatment also included amoxicillin in addition to citrus flavonoids due to the historical sanitary status of the farm and ZnO treatment included more antibiotic molecules following their conventional strategy to reduce postweaning health disorders. Although the major function of antibiotics is to act as antimicrobial substances against bacteria modifying the normal microbial community of the intestine, their anti-inflammatory effects have been also described [22]. Consequently, the effects of the ZnO and FLAV groups are discussed. However, the potential effects of all substances included in each strategy should be considered and the effects observed cannot be attributed only to zinc oxide or citrus flavonoid supplementation even if some parts of the discussion are focused on the potential effects of the supplementation of these substances.

Weaning, in addition to activating stress signaling pathways, can also alter the normal expression of intestinal genes in pigs [23,24]. After seven days of feeding, the FLAV treatment increased the jejunum mRNA levels of the genes related to barrier function (MUC13), digestive enzymes (DAO1, GCG, HNMT, and SI), and nutrient transport (SLC13A1 and SLC15A1) compared to those fed the CON diet. Citrus flavonoids, such as naringenin, possess well-demonstrated properties as anti-inflammatory and antioxidant molecules [8,25,26] that would explain the reduction in the inflammatory and antioxidant response in the intestines of these pigs. Interestingly, de Groot et al. [27] performed a study to establish inflammation patterns in weanling pigs to better understand, in future research, which interventions can positively affect the intestine in this phase. Thus, their results showed that the gene expression of proinflammatory cytokines was increased in the jejunum, ileum, and colon after weaning and during the first 15 days postweaning, for example, the expression of the IL-8 gene. Therefore, the downregulation of the IL-8 gene found in FLAV pigs, but not in the CON group, can be associated with a lower inflammatory environment in the jejunum at day seven. Indeed, citrus flavonoids’ properties improving barrier function have been largely described in humans and animals, mainly attributed to the hesperetin and naringenin flavonoids, which can regulate the expression and secretion of mucins [8]. For instance, MUC13, a transmembrane mucin glycoprotein, is an important component of intestinal structure and whose unsuitable expression may predispose to infectious and inflammatory diseases [28]. In the same way, a greater mRNA expression of nutrient transporters and digestive enzymes such as SI may indicate an enhanced intestinal maturity and absorptive function that likely contributed to improved digestion and growth. Interestingly, a downregulation of the Zn transporter gene SLC39A4 was found in ZnO pigs indicating negative feedback to restore zinc homeostasis [29].

One of the hypotheses of this work was that citrus flavonoids may be a suitable alternative to pharmacological doses of antibiotics and zinc oxide probably led by the effect of bitter compounds on bitter taste receptors (TAS2R). These taste receptors have been described along the gastrointestinal tract constituting the chemosensory system, modulating the proper digestive response (secretion, motility, absorption of nutrients, or aversion), food intake, and behavior [30,31,32,33]. In the present study, FLAV and ZnO pigs had greater expressed TAS2R39 than CON animals in the intestine at day seven, whereas ZnO increased the gene expression of TAS2R16 as well. Recently, it has been demonstrated that different TAS2Rs differentially modulate food intake in rats through different enteroendocrine secretions [34]. In accordance with these results, FLAV and ZnO pigs showed a higher ADFI at the end of ST and for the whole study (days 0 to 35). Additionally, activation of TAS2R in the stomachs of rats has been proven to stimulate ghrelin secretion, an orexigenic hormone that enhances gastrointestinal motility [35] that could explain the increase in the ADFI of pigs when zinc oxide or citrus flavonoids were used in our study. Unfortunately, the gene expression of the TAS2Rs was not analyzed in the stomachs of the pigs, so further research will be needed to elucidate if these receptors are involved in the orexigenic effect observed in FLAV and ZnO animals. Moreover, ZnO treatment also increased gene expression of TAS2R16, whereas FLAV pigs showed numerically higher gene expression than CON, though not statistically different. Paniagua et al. [9,36], observed a modulation in the gene expression of TAS2R16 in the rumen epithelium when bulls were supplemented with citrus flavonoids. Therefore, in agreement with these results, bitter substances increased the gene expression of TAS2R16 in the intestine of pigs. Although some antimicrobial molecules, such as chloramphenicol and erythromycin, have been described as bitter molecules activating TAS2R [37], as far as we know, the antibiotics used in our study have not been confirmed as bitter molecules able to activate TAS2Rs. Consequently, zinc oxide and citrus flavonoid supplementation might explain the activation of TAS2Rs observed in the intestines of the pigs.

No statistical interaction was found for treatment and tissue (jejunum and ileum) when TAS2Rs’ gene expression was analyzed, however, the gene expressions of TAS2R16 and TAS2R39 also showed different expressions depending on the tissue, jejunum, or ileum, whereas TAS2R7 and TAS2R38 were not affected. Colombo et al. [38], studied the expression of some TAS2Rs in the gastrointestinal tract of five young pigs, and they found differences in different gastrointestinal points (stomach, jejunum, and colon), and concluded that the expression for all TAS2Rs studied was very low. Genes such as TAS2R7 were predominantly found in the stomach, TAS2R16 in the jejunum and TAS2R38 was not frequently expressed. Thus, the results of the present study might be considered in agreement with Colombo et al. [38], as TAS2R7 evinced low gene expression in the intestines of all the animals; TAS2R16′s expression was found in the jejunum of the animals studied by Colombo et al. [38], and our results evinced that this receptor responded to the treatment in the tissues studied; probably TAS2R38 is not important in the gastrointestinal tract of the pig, based on the results of both studies; finally, Colombo et al. [38] did not study the TAS2R39, whereas our results have evinced that this receptor showed a higher degree of gene expression and response to the treatments. As far as we know, this is the first study in pigs where the gene expression of TAS2R39 and its response to bitter molecules have been described. Although much more research is needed to properly elucidate the functions and responses of TAS2R39 in pigs, this receptor might be involved in the multifactorial mechanism of action of zinc oxide and citrus flavonoids observed in pigs at weaning. Furthermore, TAS2R activation is related to the secretion of some enteroendocrine molecules, such as PYY and CKK [9,30,34,36]. Interestingly, CCK is an intestinal peptide that increases the release of digestive enzymes from the pancreas in the duodenum, facilitating digestion [30]. Thus, citrus flavonoids might increase digestive enzymes in the jejunum through the activation of TAS2Rs. Additionally, citrus flavonoids exert different positive effects on intestinal health through different biological mechanisms (anti-inflammatory, antioxidant, and barrier function improvement) [8].

Among all parameters that can influence animal growth performance, intestinal integrity, along with a stable microbiota, is particularly important to enhance feed efficiency and maintain overall health. Curiously, when intestinal morphology was evaluated, no differences were observed between experimental diets. However, taking a deep look into gut health parameters, diets offered to pigs modulate the microbiome community composition. Although alpha diversity indices showed a similar richness within experimental diets, the beta diversity analysis showed dissimilarities among those microbial communities. This finding suggests that the difference is not due to the presence of specific bacteria but to the larger abundance of certain bacteria [39]. Indeed, pigs fed with the ZnO diet decreased the relative abundance of genera such as *Lactobacillus* and *Lachnospira* compared to the CON diet. Interestingly, FLAV diets increased the abundance of these same bacteria genera together with *Roseburia* compared to the control diet. The importance of the intestinal microbiota for gastrointestinal function and their association with animal health and growth has been previously shown [39,40,41,42]. Indeed, several bacteria such as species of the *Lactobacillus*, *Bifidobacterium*, *Lachnospira*, or *Roseburia* genera are known as beneficial functional microbes which have great potential to contribute to the reduction of the growth of pathogenic bacteria, stimulation of the immune system, feed intake, and feed efficiency [43]. Furthermore, many members of these bacteria can produce, directly or indirectly, certain metabolites known as postbiotics, such as short-chain fatty acids (SCFA). The SCFAs (i.e., butyrate, propionate, and acetate) are produced by fermenting dietary nondigestible carbohydrates [44]. It is widely accepted that the role of SCFA as a colonocyte energy supply which instead can exert beneficial effects on intestinal barrier function and reduction of gut inflammation [45], as well as a crucial physiological effect on several organs, including the brain [46]. Therefore, these findings support the idea that FLAV treatment might improve pig growth by maintaining a stable and healthy intestinal microbiota able to increase its absorptive capacity.

Moreover, it is worth noting the lower plasma concentration of cortisol found at day seven postweaning in pigs fed the FLAV diet. Cortisol, in addition to being one of the widest biomarkers of stress in pigs, is considered a powerful suppressor of the immune response (production of cytokines and immunoglobulins) and whose secretion predominates under the prolonged exposition to a stressful stimulus [47]. As mentioned before, the stress in pigs, as a consequence of weaning, can severely impact the body‘s homeostasis and consequently, performance. Therefore, there is a primary interest in minimizing stress levels by improving management activities or through dietary interventions. The present results suggest that the FLAV diet can reduce the stress levels in pigs by approximately twofold compared to the ZnO diet, at least after the first seven days postweaning. Previous studies conducted in beef cattle have shown that supplementing citrus flavonoids can reduce aggressiveness by modulating animal behavior [9,37,48,49], so citrus flavonoids supplemented in the FLAV treatment might explain this reduction of cortisol in plasma. Nonetheless, further research is needed to corroborate the possible positive effects of citrus flavonoids on animal behavior and stress reduction in pigs, as well as the mechanisms of action associated.

In the present study, pigs fed the FLAV diet showed improved performance during the first 35 days postweaning compared to the CON diet, whereas the ZnO pigs were intermediate. Interestingly, ZnO negatively affected the abundance of some beneficial genera, such as *Lactobacillus* and *Lachnospira*, whilst only a few nutrient transporter genes were better expressed in the ilium of these animals compared with the CON piglets. On the contrary, as previously discussed, the FLAV piglets evinced better intestinal maturity together with a greater microbiome gut community, increasing the relative abundance of some genera known as beneficial functional bacteria (*Lactobacillus* or *Roseburia*), which might explain the greater performance observed in the FLAV animals at the end of the study. Consequently, although the ZnO group evinced greater ADFI, only numerical improvement was observed for BW and ADG at 35 days postweaning compared with the CON animals, whereas the FLAV piglets achieved greater BW, ADFI, and ADG.

## 5. Conclusions

Taking these results together, it must be stressed that feed and its associated nutritional components influence directly the major components of gut health such as gene expression, microbiota environment, and intestinal integrity. Consequently, supplementing feed with functional additives such as citrus flavonoids together with antibiotics like amoxicillin can positively impact intestine digestive and immune function and, thereby, on pig growth along with lesser inflammatory and pro-oxidative status, representing thus a first step to minimize the use of antibiotics and avoiding pharmacological doses of zinc oxide. Additionally, TAS2R seems to be involved in the multifactorial mechanism of action of these substances (zinc oxide and citrus flavonoids) supplemented with antibiotics. The next steps should be to evaluate the effect of the supplementation of these flavonoids without any dietary antibiotics added to confirm them as a promising alternative to dietary antibiotic use as well, improving health and performance in weanling pigs.

## Figures and Tables

**Table 1:** Composition of the basal diet, as-fed basis, for prestarter (PS) and starter (ST) phases.

Ingredients, %	PS	ST
Wheat	16.27	40.60
Maize	18.87	20.00
Acid milk whey	13.00	-
Extruded soybean meal	9.38	-
Barley	8.81	15.38
Extruded wheat	7.83	-
Extruded maize	5.22	-
Soybean meal 47%	4.05	12.98
Soybean meal concentrate	4.00	-
Fishmeal 70%	4.00	5.00
Spray Died Animal Plasma	3.00	-
Lard	2.90	2.48
Di-calcium phosphate	0.96	1.40
Calcium carbonate	-	0.11
Lysine sulfate	0.53	0.85
DL-Methionine	0.22	0.16
L-Threonine	0.18	0.24
L-Valine	0.11	-
L-Tryptophan	0.07	0.05
Sodium Chloride (Salt)	0.20	0.35
Vitamin mineral premix ^1^	0.40	0.40
		
Calculated composition, %		
Dry matter	90.10	89.50
Net energy, kcal/kg	2460	2401
Crude protein	20.70	17.99
Neutral detergent fiber	6.74	10.31
Ether extract	3.91	4.77
Ca	0.63	0.60
Total P	0.65	0.68
Dig P	0.36	0.40
		
Analyzed composition, % ^2^		
Dry matter	91.04	90.54
Crude protein	19.03	18.65
Neutral detergent fiber	7.83	9.35
Ether Extract ^2^	5.91	5.46
Ash	5.55	4.42

^1^ Provided per kg of feed: vitamin A (acetate): 7000 IU; vitamin D3 (cholecalciferol): 500 IU; vitamin D (25-hydroxicholecalciferol): 250 IU; vitamin E: 45 mg; vitamin K3: 1 mg; vitamin B1: 1.5 mg; vitamin B2: 3.5 mg; vitamin B6: 1.75 mg; vitamin B12: 0.03 mg; D-pantothenic acid: 8.5 mg; niacin: 22.5 mg; biotin: 0.1 mg; folacin: 0.75 mg; Fe (chelate of amino acid): Cu (sulfate): 2.5 mg; Cu (chelate of glycine): 7.5 mg; 20 mg; Zn (oxide): 40 mg; Zn (chelate of amino acids): 12.5 mg; Mn (oxide): 12.5 mg; Mn (chelate of glycine): 0.35 mg; I (calcium anhydrous): 0.35 mg; Co (sulfate): 0.05 mg; Se (sodium): 0.1 mg; Se (yeast): 0.5 mg. ^2^ Ether extract, HCl hydrolysis.

**Table 2:** Effect of experimental diets on growth performance of weaned pigs ^1^.

Item ^2^	Experimental Diet			SEM	*p*-Value
	CON	ZnO	FLAV		
BW, kg					
d 0	5.66	5.67	5.66	0.741	0.336
d 7	6.08	6.20	6.27	0.744	0.097
d 14	7.60	7.86	7.88	0.870	0.270
d 35	15.61 ^b^	16.44 ^a,b^	17.33 ^a^	1.446	0.010
					
ADFI, g					
d 0 to 7	107.95	104.81	115.79	9.696	0.585
d 7 to 14	318.53	333.04	301.92	18.725	0.487
d 14 to 35	496.02 ^b^	576.50 ^a^	592.38 ^a^	41.556	0.002
d 0 to 35	381.75 ^b^	437.07 ^a^	439.93 ^a^	25.813	0.001
					
ADG, g					
d 0 to 7	67.40	67.46	79.14	4.265	0.126
d 7 to 14	217.57	250.63	229.78	19.586	0.391
d 14 to 35	381.12 ^b^	408.42 ^a,b^	450.17 ^a^	28.424	0.005
d 0 to 35	284.07 ^b^	307.25 ^a,b^	333.43 ^a^	20.799	0.010
					
FCR					
d 0 to 7	1.59	1.56	1.46	0.071	0.180
d 7 to 14	1.52	1.33	1.34	0.128	0.442
d 14 to 35	1.31	1.41	1.32	0.043	0.218
d 0 to 35	1.35	1.42	1.32	0.038	0.191

^a,b^ means within a row with different superscripts are different (*p* < 0.05). ^1^ Data are means of 6 pens with 14 pigs per replicate pen. ^2^ Body weight, BW; Average daily gain, ADG.

**Table 3:** Effect of experimental diets on jejunum relative gene expression of weaned pigs fed experimental diets.

Item	Gene	Experimental Diets			Sem	*p*-Value	FDR
		CON	ZnO	FLAV			
Barrier function							
Mucin 13	*MUC13*	−0.007 ^a^	−0.067 ^a^	0.186 ^b^	0.070	0.049	0.139
Mucin 2	*MUC2*	0.044 ^a,b^	−0.175 ^b^	0.239 ^a^	0.089	0.018	0.077
Occludin	*OCLN*	0.230	0.164	0.345	0.051	0.064	0.153
Trefoil factor 3	*TFF3*	−0.061 ^a,b^	−0.261 ^b^	0.142 ^a^	0.077	0.008	0.063
Enzymes/Hormones							
Diamine oxidase	*DAO1*	0.282 ^b^	0.339 ^b^	0.568 ^a^	0.058	0.008	0.063
Glucagon	*GCG*	−0.096 ^b^	−0.104 ^b^	0.065 ^a^	0.034	0.005	0.063
Glutathione peroxidase 2	*GPX2*	0.144	−0.316	−0.159	0.119	0.054	0.139
Histamine N-methyltransferase	*HNMT*	0.168 ^b^	0.250 ^a,b^	0.322 ^a^	0.038	0.040	0.139
Sucrase-isomaltase	*SI*	0.250 ^b^	0.338 ^a,b^	0.624 ^a^	0.081	0.013	0.077
Nutrient transport							
Solute carrier family 13 (sodium/sulfate symporters) member 1	*SLC13A1*	−0.062 ^b^	0.223 ^a,b^	0.289 ^a^	0.080	0.020	0.077
Solute carrier family 15 (oligopeptide transporter) member 1	*SLC15A1*	0.586 ^b^	0.712 ^a,b^	0.902 ^a^	0.083	0.050	0.139
Solute carrier family 39 (zinc transporter) member 4	*SLC39A4*	0.154 ^a^	−0.335 ^b^	0.388 ^a^	0.082	<0.0001	0.003
Solute carrier family 5 (sodium/glucose cotransporter) member 1	*SLC5A1*	0.160 ^a,b^	0.056 ^b^	0.525 ^a^	0.108	0.019	0.077
Stress							
Glucocorticoid receptor	*NR3C1*	−0.161	−0.215	−0.074	0.064	0.304	0.471

^a,b^ means within a row with different superscripts are different (*p* < 0.05). Data are means of 6 replicate pens with 1 sampled pig per experimental diet.

**Table 4:** Effect of experimental diets on ileum relative gene expression of weaned pigs fed experimental diets.

Item	Gene	Experimental Diet			SEM	*p*-Value	FDR
		CON	ZnO	FLAV			
Barrier function							
Mucin 2	*MUC2*	−0.014 ^a^	−0.327 ^b^	−0.434 ^b^	0.066	0.001	0.031
Trefoil factor 3	*TFF3*	−0.142 ^a^	−0.390 ^a,b^	−0.658 ^b^	0.094	0.007	0.053
Immune Response							
Interferon gamma receptor 1	*IFNGR1*	−0.005 ^a^	−0.132 ^a,b^	−0.313 ^b^	0.058	0.009	0.053
Peroxisome proliferative activated receptor gamma, coactivator 1 alpha	*PPARGC1α*	−0.063 ^b^	0.260 ^a^	0.156 ^a^	0.077	0.046	0.153
Interleukin 8	*IL8*	0.043 ^a^	−0.290 ^b^	−0.274 ^b^	0.076	0.011	0.054
Enzymes/Hormones							
Glutathione peroxidase 2	*GPX2*	0.071 ^a^	−0.348 ^b^	−0.238 ^b^	0.086	0.009	0.053
Superoxide dismutase	*SOD2m*	−0.079 ^a^	−0.170 ^a,b^	−0.254 ^b^	0.035	0.013	0.056
Nutrient transport							
Solute carrier family 11 (proton-coupled divalent metal ion transporter) member 2	*SLC11A2*	−0.034 ^a^	−0.192 ^b^	−0.055 ^ab^	0.028	0.002	0.031
Solute carrier family 39 (zinc transporter) member 4	*SLC39A4*	−0.121 ^a^	−0.560 ^b^	−0.195 ^ab^	0.106	0.026	0.099
Stress							
Glucocorticoid receptor	*NR3C1*	−0.007	−0.034	−0.018	0.068	0.958	0.958

^a,b^ means within a row with different superscripts are different (*p* < 0.05). Data are means of 6 replicate pens with 1 sampled pig per experimental diet.

**Table 5:** Evenness and diversity of colon microbiota of weaned pigs fed experimental diets ^1^.

Item	Experimental Diet			*p*-Value
	CON	ZnO	FLAV	
Shannon	4.32	3.84	3.83	0.205
Simpson	0.97	0.94	0.93	0.309
Inver–Simpson	29.29	21.42	24.21	0.574

^1^ Data are means of 6 replicate pens with 1 sampled pig per experimental diet.

**Table 6:** Effect of experimental diets on histomorphometry measurements of weaned pigs at d7 ^1^.

Item	Experimental Diet			SEM	*p*-Value
	CON	ZnO	FLAV		
Villus height	257.50	323.17	293.00	19.620	0.094
Crypt depth	194.33	178.17	181.67	11.796	0.606
Ratio V:C	1.36	1.92	1.76	0.155	0.059
Intraepithelial lymphocytes	6.92	9.65	6.93	1.205	0.150
Globet cells	5.22	5.37	4.30	0.734	0.553
Mitosis	0.42	0.43	0.62	0.091	0.269

^1^ Data are means of 6 replicate pens with 1 sampled pig per experimental diet.

**Table 7:** Effect of experimental diets on proinflammatory and stress biomarkers of weaned pigs ^1^.

Item	Experimental Diet			SEM	*p*-Value
	CON	ZnO	FLAV		
d 7					
TNF	104.07	98.06	89.11	7.019	0.325
PigMap	0.85	1.16	1.04	0.129	0.134
Cortisol	17.24 ^a,b^	25.09 ^a^	11.23 ^b^	3.164	0.007
					
d14					
TNF	100.62	96.00	85.68	8.922	0.449
PigMap	0.85	0.87	0.65	0.144	0.431
Cortisol	19.81	18.04	18.81	3.696	0.936

^a,b^ means within a row with different superscripts are different (*p* < 0.05). Values from d0 were used as covariable for subsequent values. ^1^ Data are means of 6 replicate pens with 3 sampled pigs per experimental diet.

## Data Availability

Data sharing is not applicable to this article.
